# Dose-Dependent Effects of Black Soldier Fly Larvae Meal on Growth and Health of Common Carp

**DOI:** 10.3390/life16040595

**Published:** 2026-04-03

**Authors:** Lenuța Dîrvariu, Cristian-Alin Barbacariu, Marian Burducea, Gabriela Dumitru, Elena Todirascu-Ciornea, Dana Andreea Șerban, Cristina Mihaela Rîmbu, Cristina Elena Horhogea, Mircea Lazăr, Daniel Simeanu

**Affiliations:** 1 Research and Development Station for Aquaculture and Aquatic Ecology, “Alexandru Ioan Cuza” University of Iasi, 11, Carol I Blvd., 700506 Iasi, Romania; lenuta.dirvariu@uaic.ro (L.D.); dana.serban@uaic.ro (D.A.Ș.); 2 Faculty of Food and Animal Sciences, “Ion Ionescu de la Brad” Iasi University of Life Sciences, Mihail Sadoveanu Alley 8, 700489 Iasi, Romania; daniel.simeanu@iuls.ro; 3 Faculty of Biology, “Alexandru Ioan Cuza” University of Iasi, 20, Carol I Blvd., 700506 Iasi, Romania; gabriela.dumitru@uaic.ro (G.D.); ciornea@uaic.ro (E.T.-C.); 4 Department of Public Health, Faculty of Veterinary Medicine, “Ion Ionescu de la Brad” Iasi University of Life Sciences, Mihail Sadoveanu Alley 8, 700490 Iasi, Romania; crimbu@uaiasi.ro (C.M.R.); cristinahorhogea@iuls.ro (C.E.H.); 5 Department of Preclinics, Faculty of Veterinary Medicine, “Ion Ionescu de la Brad” Iasi University of Life Sciences, Mihail Sadoveanu Alley 8, 700490 Iasi, Romania; mircea.lazar@iuls.ro

**Keywords:** black soldier fly larvae meal, *Cyprinus carpio*, growth performance, oxidative status, microbiota

## Abstract

The aim of this study was to evaluate the effects of incorporating Black Soldier Fly Meal (BSFLM) into the diet of two-year-old carp on growth and health status. Four concentrations of BSFLM were tested, T1-0% (control), T2-10%, T3-20%, and T4-30%, in common carp with an initial body weight of 112.06 ± 3.86 g over a 12-week feeding period. The results showed that final weight and weight gain were 17.3% (349.15 g) and 23% (233.6 g) higher in the T4 group compared to the control (298.63 g and 189.79 g, respectively). Flesh composition showed no significant changes. Hematological variations were insignificant. Oxidative stress assessment revealed increased activities of antioxidant enzymes (SOD, CAT, and GPx) alongside reduced lipid peroxidation. The counts of anaerobic microorganisms and Enterobacteriaceae in intestine increased linearly in BSFLM groups. Histological examination showed normal intestinal and hepatopancreatic morphology in the 10% and 20% BSFLM groups (T2 and T3). In contrast, the 30% inclusion level (T4) was associated with hepatocellular vacuolization and alterations in villus architecture. In two-year-old carp, dietary BSFLM induced dose-dependent responses, whereby higher inclusion levels supported weight gain but adversely affected the morphological integrity of the intestine and hepatopancreas.

## 1. Introduction

Aquaculture is a key contributor to global food security, representing the fastest-growing sector within the food industry. According to the most recent report from the Food and Agriculture Organization of the United Nations [1], aquaculture contributed 122.6 million tons to the total global fishery and aquaculture production of 214 million tons in 2020, valued at 281.5 billion USD. Fish production in the European Union reached 1,084,408.44 tons in 2022 with cyprinid production accounting for 75,150 tons [2]. Specifically, carp production in Romania totaled 7522 tons in 2022 [2], representing a concerning linear decline in recent years that reflects troubling trends within this sector in Romania. One of the primary factors contributing to the decline in aquaculture production in Romania is the elevated cost of specialized feeds. Feed importation has become a common practice because of the shortage of essential ingredients such as fishmeal and fish oil, which are expensive and logistically challenging to obtain locally [3]. This situation compels farmers to utilize conventional feeding approaches based on cereals and by-products, resulting in elevated feed conversion ratios, extended time to reach marketable size, and reduced production per hectare. Under current global aquaculture conditions, where fishmeal sources are becoming increasingly limited while the sector continues linear growth, identifying novel protein sources is essential.

Insect use as a nutrient source is well-established, with entomophagy being a longstanding practice in regions such as Latin America, Africa, and Asia. In Europe, interest in insect protein has increased since the European Union authorized the inclusion of this ingredient in aquafeeds in 2017 [4]. According to literature data [5], insect protein emerges as the most promising fishmeal alternative. In comparison with other emerging protein sources (microalgae, macroalgae, bacteria, and yeast), insect meal—mainly from *Hermetia illucens*—shows high potential as a substitute for fishmeal [6,7]. Several studies have demonstrated that fishmeal replacement with Black Soldier Fly Larvae Meal (BSFLM) is feasible at levels ranging from 22.5% to as high as 75% in different fish species [6,8,9,10,11,12,13]. The significance of BSFLM lies in its protein content of approximately 40% [14,15] and its composition of essential amino acids and fatty acids. The principal essential amino acids in BSFLM include lysine, methionine, threonine, tryptophan, leucine, isoleucine, valine, phenylalanine, histidine, and arginine, while non-essential amino acids comprise glutamic acid, aspartic acid, proline, alanine, glycine, serine, tyrosine, and cysteine [16,17,18,19,20]. The primary saturated fatty acids (SFAs) in BSFLM include lauric acid (C12:0), myristic acid (C14:0), and palmitic acid (C16:0); monounsaturated fatty acids (MUFAs) include oleic acid (C18:1, n-9) and palmitoleic acid (C16:1); and polyunsaturated fatty acids (PUFAs) include linoleic acid (C18:2, n-6) and α-linolenic acid (C18:3, n-3) [16,20,21,22,23]. Compared to fishmeal, BSFLM exhibits lower levels of methionine, lysine, tryptophan, and omega-3 fatty acids, while protein digestibility is reduced due to the presence of chitin in the exoskeleton. However, BSFLM demonstrates higher levels of proline, glycine, tyrosine, and arginine [8,10]. In some species, particularly carnivorous fish, BSFLM can be utilized in smaller proportions relative to fishmeal or may adversely affect certain growth parameters or antioxidant capacity [24,25]. Notably, chitin from BSFLM has demonstrated positive effects on growth parameters and oxidative status [26,27]. Several studies have also evaluated the use of BSFLM oil with promising results [27,28,29]. Zhou et al. (2017) [30] reported that complete replacement of fishmeal with BSFLM did not negatively affect growth in Jian carp, although significant changes in fatty acid composition were observed, including reductions in n-3 highly unsaturated fatty acids. Similarly, Dogan et al. (2021) [31] showed improved growth performance at intermediate inclusion levels, while higher inclusion rates may induce physiological stress and intestinal alterations. More recent work, such as Albayati et al. (2025) [32], confirmed that high inclusion levels (75–100%) can enhance growth performance and feed efficiency in common carp without major hematological disturbances. In addition, Linh et al. (2024) [33] reported improved growth performance in carp species at high inclusion levels of BSFLM. However, most existing studies have primarily focused on growth performance and basic physiological responses, with limited integration of oxidative status, intestinal microbiota, and histological changes. Therefore, a comprehensive, multi-level evaluation of BSFLM inclusion remains limited, particularly in relation to dose-dependent physiological trade-offs.

Moreover, the integration of histological examination in nutritional studies provides important insights into the physiological adaptations and potential adverse effects of dietary modifications at the tissue level. In this context, the hepatopancreas, as the central organ for nutrient metabolism and detoxification, and the intestine, as the primary site of nutrient absorption, function as sensitive indicators of dietary tolerance and nutritional adequacy. Previous investigations have demonstrated the value of histomorphological evaluation for elucidating cellular responses to alternative protein sources in fish nutrition [34,35,36].

Accordingly, the aim of the present study was to evaluate the dose-dependent effects of dietary BSFLM on growth performance, health-related biochemical responses, intestinal microbiota, oxidative status, and organ morphology in two-year-old common carp, in order to determine the suitability of BSFLM as a sustainable protein source for carp aquaculture.

## 2. Materials and Methods

### 2.1. Fish Husbandry and Rearing Conditions

For this study, 144 common carp (*Cyprinus carpio*) specimens were used. Fish were two summers old and had an average weight of 112.06 ± 3.86 g. Fish were selected from a stock belonging to the Research and Development Station for Aquaculture and Aquatic Ecology. Prior to the trial, experimental fish were transferred to a recirculating aquaculture system (RAS) and acclimated for two weeks. Individuals were then randomly distributed among 12 tanks (1 m^3^ each) with 12 individuals per tank, resulting in 36 fish per dietary treatment. Tanks were randomly assigned to treatments (diet). The feeding trial lasted 12 weeks. All fish specimens were measured and weighed at the beginning of the experiment and at every three weeks. Throughout the experimental period, fish health status, feeding behavior, and mortality were monitored daily. Fish were hand-fed the experimental diets daily at 08:00, 12:00, and 15:00. The daily feed ration was set at 5% of body weight and was adjusted according to water temperature and changes in fish biomass. Every three weeks, the fish were weighed to calculate growth parameters and to adjust the feeding ration; additionally, between weighing events, the ration was increased by 10% every 10 days. This percentage is optimal for this life stage at temperatures between 20 and 25 °C. No mortalities were recorded during the experimental period.

Water physicochemical parameters were continuously monitored to maintain optimal conditions for carp culture. Dissolved oxygen, temperature, and pH were measured daily before feeding using Hach HQ1130 and Hach HQ11D meters (Hach Lange S.R.L., Bucharest, Romania), respectively. Additional parameters—including ammonia, ammonium, nitrate, nitrite, total hardness, calcium, magnesium, and iron—were determined weekly with a Hanna Iris HI801 spectrophotometer (Hanna Instruments Service S.R.L., Cluj-Napoca, Romania). During the experiment, water temperature ranged from 20.5 to 25.2 °C, pH from 7.8 to 8.0, and dissolved oxygen from 7.99 to 8.65 mg L^−1^. Unionized ammonia concentrations remained between 0.00 and 0.05 mg NH_3_-N L^−1^. Detailed water quality data are presented in Table 1.

### 2.2. Experimental Diets

The experimental diets were formulated to include four graded levels of Black Soldier Fly Larvae Meal (BSFLM): T1 (0% BSFLM), T2 (10% BSFLM), T3 (20% BSFLM), and T4 (30% BSFLM). Each treatment was conducted in triplicate. Feed ingredients comprised meat meal, fish meal, soybean meal, corn, wheat, distillers dried grains with solubles (DDGS), peas, soybean oil, and BSFLM. All ingredients were sourced from Romania, except for fish meal.

Dietary components were weighed, thoroughly mixed, ground, extruded, and pelleted. The proximate composition of ingredients and experimental diets was determined following [38] standard procedures. Moisture content was measured by oven drying at 105 °C (Nahita 631/6, AUXILAB, S.L., Beriáin, Spain). Crude lipid content was determined with Soxhlet extraction (Behr Behrotest RFK 100, Behr Labor-Technik GmbH, Düsseldorf, Germany) using ether as solvent. Crude protein was quantified by the Kjeldahl method (Kjeltec™ 8100, FOSS, Hillerød, Denmark) based on total nitrogen content. Crude fiber was calculated as the loss on ignition of the dry residue obtained after sequential digestion with 1.25% (*w*/*v*) H_2_SO_4_ and 1.25% (*w*/*v*) NaOH, in accordance with standard analytical protocols (Table 2).

The amino acid profile of BSFLM was analyzed by high-performance liquid chromatography (HPLC) using a Shimadzu LC-10ADVP, Columbia, MD, USA, following SR EN ISO 13903:2005 and European Commission Regulation (EC) No. 152/2009 (Table 3). Fatty acid composition was determined after conversion of lipids to fatty acid methyl esters (FAMEs) according to SR CEN ISO/TS 17764-1:2008, followed by gas chromatographic analysis with flame ionization detection (Perkin Elmer system, PerkinElmer, Shelton, CT, USA) using an appropriate capillary column, in accordance with SR CEN ISO/TS 17764-2:2008 and SR EN ISO 5508:2002 (Table 4). Proximate composition of feeds is reported in Table 5.

**Table 3:** Amino acid profile of the Black Soldier Fly Larvae Meal (g kg^−1^).

Amino Acids	(g kg^−1^)
Glutamic acid	47.55
Aspartic acid	31.25
Alanine	26.44
Leucine	27.37
Lysine	23.06
Valine	23.16
Proline	22.65
Glycine	20.90
Arginine	19.78
Tyrosine	24.70
Serine	17.42
Phenylalanine	15.58
Threonine	15.06
Isoleucine	16.70
Histidine	10.76
Methionine	5.84
Cysteine	2.97

**Table 4:** Composition of fatty acids of the Black Soldier Fly Meal (g kg^−1^).

Fatty Acids	(g kg^−1^)
Lauric acid (C12:0)	21.00
Palmitic Acid (C16:0)	3.54
Miristic acid (C14:0)	2.65
Oleic acid (C18:1)	2.71
Linoleic acid (C18:2)	1.73
SAFA	17.73
MUFA	3.84
PUFA	1.97
Omega-3	0.15
Omega-6	1.73

**Table 5:** Diet composition for carp.

Parameter	T1 (0% BSFLM)	T2 (10% BSFLM)	T3 (20% BSFLM)	T4 (30% BSFLM)
Ingredient composition (g kg^−1^)				
Meat meal ^1^	350	250	150	50
Sunflower meal	260	260	260	260
Fish meal ^2^	150	150	150	150
Soybean meal	100	100	100	100
BSFLM ^3^	0	100	200	300
Peas	50	50	50	50
DDGS ^4^	50	50	50	50
Soybean oil	30	30	30	30
Vitamin premix ^5^	10	10	10	10
Chemical composition of experimental diets (%)				
Moisture	10.33	10.06	10.01	10.15
Crude protein	31.84	31.13	31.88	31.71
Crude fat	7.74	8.09	8.08	8.83
Crude fiber	7.47	7.60	7.20	7.82

^1^ Meat meal producer: Safir, Romania. ^2^ Fishmeal producer: C.A.N. 2000 Trading SRL, Romania. ^3^ Black Soldier Fly Meal producer: DK Agroshop SRL, Romania. ^4^ Dried distillers grains with solubles. ^5^ Vitamin A—50,000 IU, Vitamin D_3_—5000 IU, and Vitamin E—20 mg.

### 2.3. Evaluation of Growth Performance, Feed Utilization Efficiency and Physiological Indices

Growth indicators were determined by three measurements of fish length (cm) and weight (g):

Initial body weight (IBW, g) Final body weight (FBW, g) Weight gain (WG, g) = FBW − IBW Specific growth rate (SGR, % day^−1^) = [(ln FBW − ln IBW)/days of experiment] × 100 RGR-relative growth rate (RGR, g g^−1^ day^−1^) = WG/(IBW × experimental days) Feed conversion ratio (FCR) = feed intake/WG Protein efficiency ratio (PER) = WG/protein intake (g) Lipid efficiency ratio (LER) = WG/lipid intake (g) Condition factor (CF) = (FBW/body length^3^) × 100 Hepatosomatic index (HSI, %) = (final liver weight/FBW) × 100 Viscerosomatic index (VSI, %) = (final visceral weight/FBW) × 100

### 2.4. Proximate Composition of Fish Flesh

To obtain the flesh samples required for analysis, fish were euthanized using a solution of 2% clove essential oil. The proximate composition of the muscle tissue, obtained from 10 sample collected from 5 fish for each treatment (moisture, protein, lipid, ash, collagen, and salt), was determined using an automated near-infrared analyzer DA 7250 (Perten Instruments, Hagersten, Sweden). Each sample required only 6 s to be analyzed. The analyzer is factory-precalibrated for all types of meat products based on more than 1500 samples analyzed using standard reference methods. In addition, the calibration is periodically verified for accuracy in fish samples to ensure the reliability of the measurements.

### 2.5. Blood Hematological Profile

For hematological profiling, blood samples were collected by caudal puncture. The collection was done in EDTA (ethylenediaminetetraacetic acid) tubes, and the obtained samples were analyzed using a Mindray BC-5000 Vet automatic hematology analyzer (Shenzhen Mindray Animal Medical Technology Co., Ltd., Shenzhen, China). Before blood collection, the fish were anesthetized in a 0.2% clove oil solution for 3 min. The parameters measured included: leukocytes, lymphocytes, monocytes, neutrophils, eosinophils, basophils, red blood cells, hemoglobin, hematocrit, mean corpuscular volume, platelets, mean platelet volume, and platelet thrombocrit.

### 2.6. Oxidative Status

For the assessment of the oxidative status, tissue samples of muscle, liver and intestine were obtained from four specimens per treatment. The collected tissues were homogenized in a cold potassium phosphate-buffered solution (0.1 M, containing 1.15% KCl, pH 7.4), in a ratio of 1:10 (*w*/*v*), and centrifuged for 20 min at 3000 rpm and 4 °C. The supernatants were used to determine the activities of superoxide dismutase, catalase and glutathione peroxidase, as well as to quantify the level of malondialdehyde (MDA), according to the methodology described by [39]. Antioxidant enzyme activities and MDA concentrations were normalized according to the total soluble protein content, determined by [40].

### 2.7. Intestinal Microbiota

The intestinal contents were obtained from the same intestinal segment (mid-intestine) in all fish under aseptic conditions, weighed (1 g), and transferred to sterile vials. Microbiological analysis aimed to evaluate quantitative variations in microbiological indicators in order to highlight changes in the intestinal microbiota caused by dietary interventions. The microbiological groups determined as colony-forming units (CFUs) per gram of intestinal content were: (i) total aerobic bacteria (CFU/g), (ii) total anaerobic bacteria (CFU/g), (iii) sulfite-reducing Clostridia (CFU/g), and (iv) Enterobacteriaceae (CFU/g). For statistical interpretation, CFU/g was transformed into log_10_ CFU/g.

The methodology followed procedures described by Barbacariu et al. (2021) [39]. Thus, decimal dilutions (10^1^–10^3^) were prepared from one gram of intestinal content. From each dilution, 1 mL was distributed into sterile Petri dishes, to which specific culture media corresponding to each microbiological indicator were added. To determine the total aerobic bacteria, Plate Count Agar (PCA, Dialab, Wiener Neudorf, Austria) was used, incubated at 25 °C for 24 h under aerobic conditions. To evaluate total anaerobic bacteria, the plates were incubated at 25 °C for 48 h under anaerobic conditions. Sulfite-reducing Clostridia were determined using sulfite–polymyxin B–sulfadiazine agar (SPS Agar, Oxoid Ltd., Basingstoke, Hampshire, UK), incubated at 37 °C for 48 h under anaerobic conditions. Enterobacteriaceae were determined using Bromocresol Purple Lactose Agar (BCP, Dialab) chromogenic medium, incubated at 37 °C for 48 h under aerobic conditions.

### 2.8. Histopathological Examination

The tissue fragments of intestine and hepatopancreas were fixed in 10% buffered formalin solution. The tissues were paraffin-embedded with the Leica TP1020 automatic tissue processor (Leica Microsystems GmbH, Wetzlar, Germany). Sections (5 µm) were cut using a SLEE CUT 6062 microtome, then deparaffinized and stained with Masson’s trichrome. (SLEE Medical GmbH, 246, Wetzlar, Germany). Qualitative histological analysis was carried out on stained sections using the MoticEasyScan Pro 6 digital slide scanning system (Motic Europe, Cabrera de Mar, Barcelona, Spain), a high-capacity device enabling the simultaneous automated digitization of multiple glass slides and providing an integrated platform for pathological evaluation. The slides were scanned at high resolution, and the images were automatically archived on the dedicated slide management server. The histological examination was aimed at on evaluating the structural organization, cellular morphological characteristics, and any pathological changes in the tissues.

### 2.9. Statistical Analysis

Before statistical analysis, data distribution was evaluated for normality using the Shapiro–Wilk test and for homogeneity of variances (e.g., Levene’s test). Growth performance and physiological parameters (IBW, FBW, WG, SGR, PER, LER, CF, HSI, and VSI) were further evaluated using orthogonal polynomial contrasts to determine linear and quadratic response trends. All measured variables, including meat proximate composition, blood biochemical profile, and intestinal microbiota, were analyzed by one-way analysis of variance (ANOVA), followed by Tukey’s post hoc test for multiple comparisons in SPSS version 21 (IBM Corp., Armonk, NY, USA). Statistical significance was set at *p* < 0.05 and results are presented as means ± standard error of the mean (SEM).

Biochemical indicators (oxidative stress markers) were analyzed separately by one-way ANOVA followed by Tukey’s multiple comparison test (*p* < 0.05) using GraphPad Prism version 9.3.1 (GraphPad Software, La Jolla, CA, USA).

## 3. Results

### 3.1. Evaluation of Growth Performance Feed and Physiological Indices

Growth parameters and feed utilization indices of two-year-old carp fed diets containing different levels of Black Soldier Fly Larvae Meal (BSFLM) are presented in Table 6. Initial body weight (IBW) did not differ significantly among treatments (*p* = 0.05), ranging from 107.84 ± 2.79 g in the control (T1) to 115.56 ± 2.04 g in T4 (30% BSFLM). In contrast, final body weight (FBW) was significantly affected by dietary inclusion (*p* = 0.001). Fish in T4 reached 349.15 ± 10.79 g, representing a 17.3% increase compared to the control (297.63 ± 10.42 g). Similarly, weight gain (WG) increased significantly with diet (*p* = 0.001), with T4 achieving 233.6 ± 13.86 g, a 23% increase relative to T1. Both FBW and WG showed significant linear (*p* = 0.001) and quadratic responses (FBW *p* = 0.003; WG *p* = 0.005) to increasing BSFLM levels. In line with these results, specific growth rate (SGR) was also significantly higher in T4 (4.36 ± 0.11% day^−1^) compared to the control (4.01 ± 0.08% day^−1^; *p* = 0.006). On the other hand, relative growth rate (RGR) did not differ significantly among treatments (*p* = 0.72). Regarding feed utilization, feed conversion ratio (FCR) was not significantly affected by diet (*p* = 0.307), although a slight decrease was observed with increasing BSFLM levels (from 2.28 ± 0.08 in T1 to 2.12 ± 0.04 in T4). PER increased significantly with diet (*p* = 0.037), reaching the highest value in T3 (0.86 ± 0.02), and showed a significant linear trend (*p* = 0.03). Lipid efficiency ratio (LER) also varied significantly among treatments (*p* = 0.021) and displayed a quadratic response (*p* = 0.01), with T2 exhibiting the highest value (3.46 ± 0.09). Finally, condition factor (CF) decreased slightly with increasing BSFLM (*p* = 0.05). In contrast, HSI and VSI remained unaffected (*p* = 0.372 and *p* = 0.74, respectively).

### 3.2. Proximate Composition of Flesh

The proximate composition of two-year-old carp filets fed diets containing different levels of Black Soldier Fly Larvae Meal (BSFLM) is presented in Table 7. Moisture content varied significantly among treatments (*p* = 0.022), ranging from 74.66 ± 0.22% in the control (T1) to 76.6 ± 0.32% in T4. A gradual increase in moisture was observed with higher BSFLM inclusion, with T3 and T4 showing the highest values. Protein content did not differ significantly among treatments (*p* = 0.158), ranging from 18.4 ± 0.09% to 18.84 ± 0.18%. In contrast, lipid content was significantly influenced by diet (*p* = 0.001). The control and T2 showed the highest lipid levels (2.16 ± 0.11% and 2.3 ± 0.11%, respectively), whereas T3 and T4 exhibited marked reductions (0.92 ± 0.17% and 1.5 ± 0.13%, respectively). Ash content also varied significantly among treatments (*p* = 0.001), with T3 recording the highest ash content (3.94 ± 0.32%), while T4 had the lowest (0.88 ± 0.06%). Similarly, collagen content was significantly affected by diet (*p* = 0.001), ranging from 0.34 ± 0.07% in T4 to 1.18 ± 0.09% in T2. Finally, salt content showed a significant difference among treatments (*p* = 0.039), although variations were relatively small, ranging from 0.46 ± 0.18% in T4 to 1.06 ± 0.08% in T2.

### 3.3. Blood Hematological Examination

Hematological parameters of two-year-old carp fed diets with different inclusion levels of Black Soldier Fly Larvae Meal (BSFLM) are summarized in Table 8. Overall, no parameters showed statistically significant differences among treatments (*p* > 0.05). In the T2 and T3 groups, slight lymphopenia was associated with neutrophilia, which is more pronounced in T2 (approximately three-fold increase compared to the control group) and in T3 (approximately twice the control value). In the T4 group, slight lymphocytosis was associated with markedly reduced neutrophilia. In the T3 and T4 groups, blood monocyte counts were reduced by half compared to the control group, while T2 showed significant monocytosis. All groups treated with insect meal demonstrated a slight increase in red blood cell populations, associated with improved hemoglobin levels in the T3 group. In the T2 group, platelet numbers were reduced, while the other two treatments showed slight increases.

### 3.4. Intestinal Microbiota

**Figure 1:**
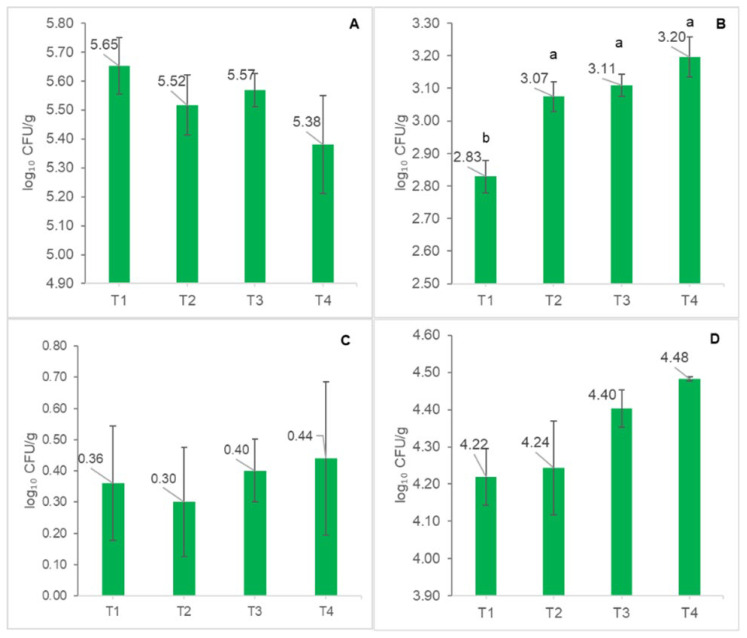
Effects of dietary inclusion of Black Soldier Fly Meal (BSFLM) at 0% (T1, control), 10% (T2), 20% (T3), and 30% (T4) on intestinal microbiological indicators in two-year-old common carp. (**A**) Total aerobic bacteria (log_10_ CFU/g), (**B**) total anaerobic bacteria (log_10_ CFU/g), (**C**) sulfite-reducing Clostridia bacteria (log_10_ CFU/g), and (**D**) Enterobacteriaceae (log_10_ CFU/g). The values are expressed as means ± S.E.M. Different lowercase letters represent statistically significant differences according to Tukey’s test at *p* < 0.05.

The intestinal microbiota was quantitatively assessed based on total aerobic bacteria, total anaerobic bacteria, sulfite-reducing Clostridia, and Enterobacteriaceae (Figure 1A–D). Total aerobic bacterial counts (Figure 1A) showed minor variation among treatments, ranging from 5.38 to 5.65 log_10_ CFU/g, with no statistically significant differences between the control (T1, 0% BSFLM) and BSFLM-supplemented groups (T2–T4). In contrast, total anaerobic bacteria (Figure 1B) exhibited a clear increasing trend with rising BSFLM inclusion. Values increased from 2.83 log_10_ CFU/g in the control to 3.07, 3.11, and 3.20 log_10_ CFU/g in T2 (10%), T3 (20%), and T4 (30%), respectively, with the control group showing significantly lower counts than the supplemented treatments (*p* < 0.05). Sulfite-reducing Clostridia (Figure 1C) increased across all dietary treatments, from 0.30 log_10_ CFU/g in the control group (T1) to 0.44 log_10_ CFU/g in T4. The abundance of Enterobacteriaceae (Figure 1D) increased progressively with BSFLM inclusion, from 4.22 log_10_ CFU/g in the control to 4.24, 4.40, and 4.48 log_10_ CFU/g in T2–T4.

### 3.5. Oxidative Stress

**Figure 2:**
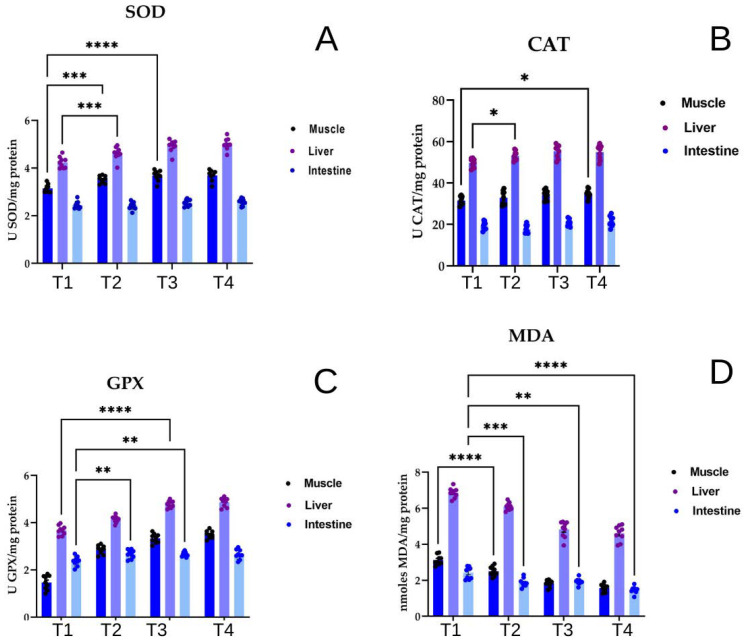
Effects of dietary inclusion of Black Soldier Fly Meal (BSFLM) at 0% (T1, control), 10% (T2), 20% (T3), and 30% (T4) on (**A**) superoxide dismutase (SOD) specific activity, (**B**) catalase (CAT) specific activity, (**C**) glutathione peroxidase (GPX) specific activity and (**D**) malondialdehyde (MDA) levels in muscle, liver, and intestinal tissues. The values are expressed as means ± S.E.M. For Tukey’s multiple comparisons analysis, **** *p* < 0.0001, *** *p* < 0.001, ** *p* < 0.01, and * *p* < 0.05.

The oxidative status of muscle, liver, and intestinal tissues in two-year-old common carp is presented in Figure 2A–D. Superoxide dismutase (SOD) activity varied depending on the investigated tissue and the amount of insect meal administered (Figure 2A). In the control group (T1), SOD activity reached 3.008 ± 0.251 U/mg protein in muscle, 3.991 ± 0.325 USOD/mg protein in liver tissue, and 2.349 ± 0.275 USOD/mg protein in intestinal samples. In experimental variants supplemented with BSFLM, increased enzyme activity was observed in direct correlation with insect meal concentration, with minimum activity recorded in the intestine, where no significant differences between experimental groups were detected.

Catalase (CAT) activity (Figure 2B) showed minimal fluctuations across experimental variants, with differences observed only between analyzed tissues. The highest values were recorded in the liver, followed by muscle and intestine.

Glutathione peroxidase (GPX) activity (Figure 2C) fluctuated in direct correlation with insect meal dosage and tissue type analyzed. In the control group (T1), average GPX activity in liver tissue was 3.419 ± 0.255 UGPx/mg protein, while in groups fed insect meal-supplemented diets, activity increased proportionally with flour concentration: 4.135 ± 0.315 UGPx/mg protein in T2, 4.612 ± 0.255 UGPx/mg protein in T3, and 4.863 ± 0.395 UGPx/mg protein in T4.

Insect meal administration decreased lipid peroxidation levels in analyzed samples (Figure 2D). In muscle tissue, the control group (T1) showed an average MDA concentration of 3.125 ± 0.553 nmoles MDA/mg protein, while T2 demonstrated a decrease to 2.505 ± 0.455 nmoles MDA/mg protein, and T4 showed approximately 50% reduction compared to T2. Similar trends were observed in intestinal tissue, with the control group recording 2.421 ± 0.522 nmoles MDA/mg protein, while in the T4 group, MDA value reached 1.472 ± 0.374 nmoles MDA/mg protein. The control group showed 6.855 ± 0.705 nmoles MDA/mg protein, T2 recorded 6.102 ± 0.575 nmoles MDA/mg protein, and T4 reached only 4.608 ± 0.785 nmoles MDA/mg protein (Figure 2D).

### 3.6. Histopathological Examination

**Figure 3:**
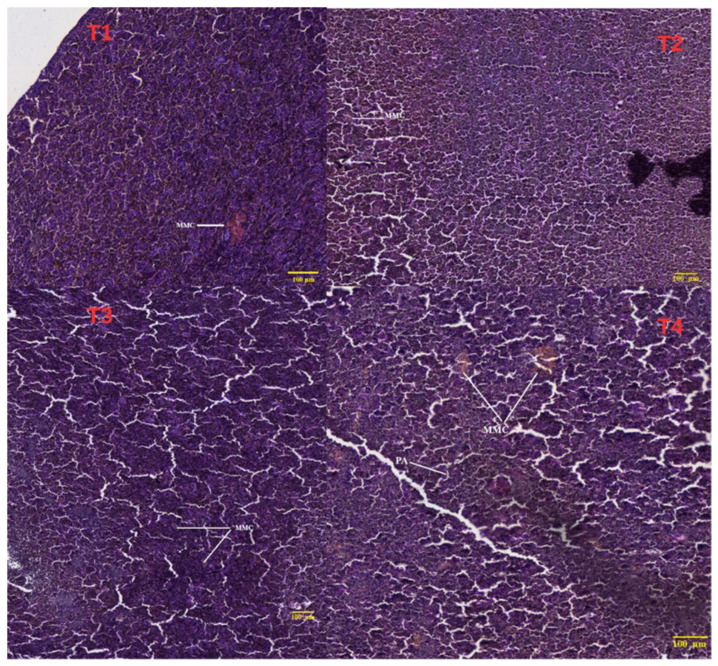
Histological examination of hepatopancreatic tissue in *Cyprinus carpio* from control group T1, T2 (10% BSFLM), T3 (20% BSFLM), and T4 (30%BSFLM) diets (Masson’s trichrome staining). Abbreviation: MMC—Melanomacrophage centers.

**Figure 4:**
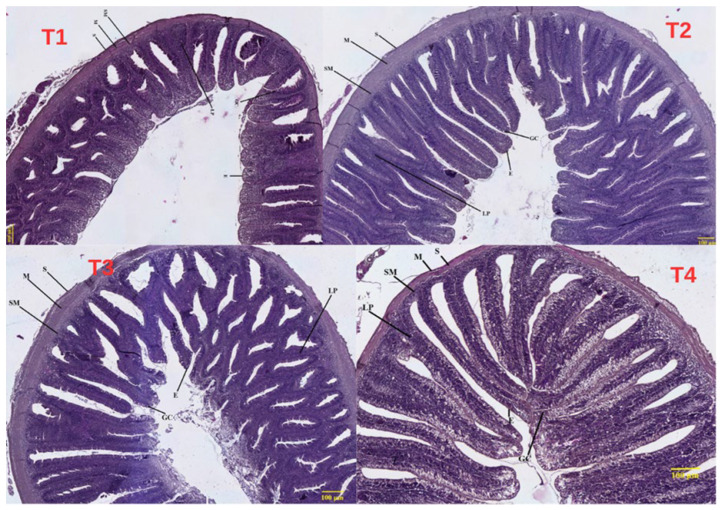
Cross-sectional histological analysis of intestinal tissue in *Cyprinus carpio* from control group T1, T2 (10% BSFLM), T3 (20% BSFLM), and T4 (30%BSFLM) diets (Masson’s trichrome staining). Abbreviations: S—serosa; M—muscularis; SM—submucosa; LP—lamina propria; E—epithelium; GC—goblet cell.

**Figure 5:**
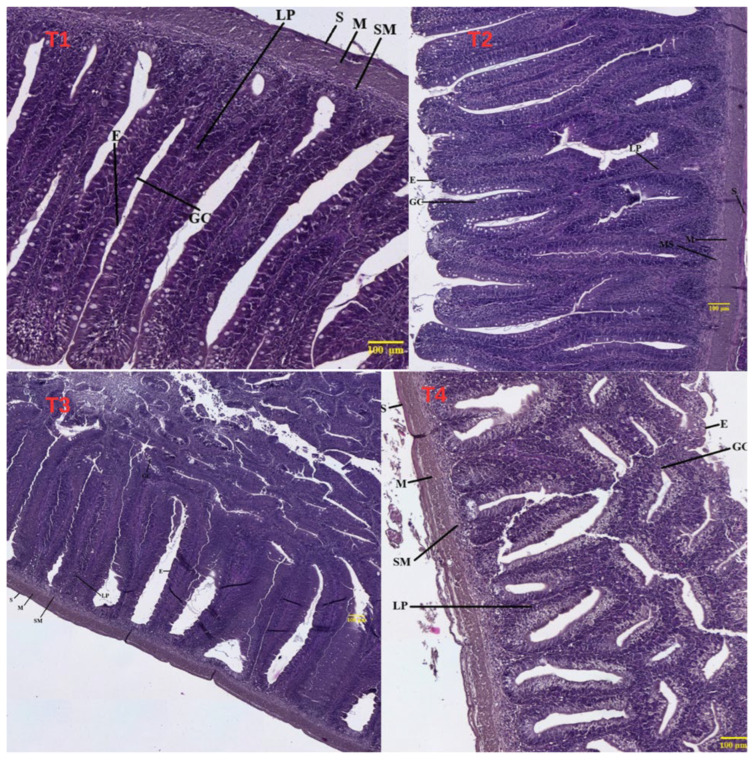
Longitudinal histological section of intestinal tissue in *Cyprinus carpio* from control group fed T1, T2 (10% BSFLM), T3 (20% BSFLM), T4 (30%BSFLM) diet, (Masson’s trichrome staining). Abbreviations: S—serosa; M—muscularis; SM—submucosa; LP—lamina propria; E—epithelium; GC—goblet cell.

Histological examination of the hepatopancreas and intestinal tissues of two-year-old common carp is presented in Figure 3, Figure 4 and Figure 5.

Control (T1 0% BSFLM): Histological examination of hepatopancreatic parenchyma in control fish revealed normal, well-organized architecture (Figure 3). Hepatocytes were arranged in radial cords separated by narrow sinusoids with regular morphology. Hepatocyte cytoplasm was moderately eosinophilic, with round, centrally located nuclei uniformly distributed. No vacuolization, degeneration, or signs of hepatic steatosis were observed. Exocrine pancreatic structures were present as well-defined acini distributed diffusely within the hepatic parenchyma, with preserved cellular polarity and zymogen granulations in the apical region. No inflammatory infiltrates or other pathological changes were present, characteristic of physiological hepatopancreatic tissue unaffected by dietary factors or metabolic stress. Intestinal examination showed long, well-individualized villi covered by simple columnar epithelium with regularly distributed goblet cells (Figure 4). The chorion was well-organized without signs of inflammatory infiltrate or edema. The muscular tunica was evident, formed by two well-demarcated layers (inner circular and outer longitudinal), while the submucosa appeared thin with reduced cellular density. No hyperplastic lesions, villous atrophy, or signs of tissue remodeling were observed, reflecting functional intestinal architecture characteristic of healthy specimens without tissue reactions suggestive of food intolerance or enteric stress.

T1 (10% BSFLM): Hepatopancreatic tissue presented a generally physiological appearance with minor changes compatible with adaptation to the new dietary regime containing 10% BSFLM (Figure 3). The absence of major lesions suggested good tolerance of this fishmeal replacement level. At the intestinal level, general morphology was well-preserved, comparable to the control group (Figure 4 and Figure 5). Intestinal villi were tall and relatively thin with radial orientation, covered by continuous simple columnar epithelium without signs of desquamation. Goblet cells were present and well-distributed along the villi, indicating normal secretory activity. Slight thickening of the chorion was noted, possibly associated with moderate immunological activity, but without evident inflammatory infiltrates. Muscular layers were well-differentiated, and the submucosa showed no edema or hemorrhage. Lieberkühn crypts were present but slightly deeper compared to controls, potentially reflecting proliferative adaptation to the new diet type.

T3 (20% BSFLM): Histological examination of hepatopancreatic tissue revealed well-conserved hepatic architecture with hepatocytes organized in regular radial cords separated by clear sinusoids (Figure 3). Hepatocyte cytoplasm was slightly granular with uniform appearance, without signs of vacuolization or degeneration. Nuclei were central and round, with well-visible nucleoli, suggesting high metabolic activity consistent with superior growth performance observations in this group. Exocrine pancreatic structures were well-represented as compactly arranged acini with preserved cellular polarity and abundant zymogen granulations in the apical region. No inflammatory infiltrates, necrosis, or vascular congestion were evident, indicating metabolically active and functionally competent hepatopancreatic tissue.

Intestinal cross-sections showed well-conserved histological organization with tall, elongated, and slightly more robust villi compared to controls (Figure 4 and Figure 5). The covering epithelium was simple columnar with regular goblet cell distribution, indicating normal mucus secretion with potential protective roles. Increased chorion density was noted with slight villous hypertrophy, without inflammatory infiltrate presence. The submucosa was well-demarcated, and muscular layers showed relatively uniform thickness without signs of hyperplasia or atrophy. Lieberkühn crypts were evident in depth, more numerous and slightly more developed compared to controls, possibly reflecting intensified regenerative and proliferative activity. These morphological characteristics may correlate with well-utilized protein intake and efficient intestinal absorption.

T4 (30% BSFLM): Histological analysis of hepatopancreatic parenchyma revealed discrete organizational changes compared to control and other treatment groups (Figure 3). Hepatocytes were arranged in relatively irregular cords, with cytoplasm showing increased granularity and focal vacuolization zones, indicating possible metabolic stress. Some sinusoids appeared slightly dilated, and hepatocyte cord delimitation was locally unclear. Hepatocyte nuclei generally maintained round shape and central position, but cells with intensely basophilic nuclei were present, suggesting increased regenerative activity. Exocrine pancreatic structures were visible but more dispersed and less compact compared to previous groups, with reduced zymogen granulation intensity.

Intestinal examination showed generally preserved architecture but with discrete morphological changes compared to other groups (Figure 4 and Figure 5). Intestinal villi were elongated with slight thickening and non-uniform chorion thickness variation, with uneven goblet cell distribution. Simple columnar epithelium remained continuous, but focal hyperplasia and villous height discrepancies were observed in certain areas. Lieberkühn crypts were present but wider and more superficial compared to previous groups, potentially reflecting increased compensatory proliferative activity, suggesting adaptive reaction or mild mucosal stress. The submucosa and muscular layer were well-demarcated without signs of evident edema or inflammation. Longitudinal sections revealed shortened and thickened villi with slightly irregular contours and more compact general architecture. Epithelial covering remained columnar but showed non-uniform goblet cell distribution with apparently reduced density in certain zones. Lieberkühn crypts were markedly developed and deeper, potentially reflecting increased compensatory proliferative activity.

## 4. Discussion

The objective of this study was to determine the effects of Black Soldier Fly (*Hermetia illucens)* Larvae Meal (BSFLM) utilization in *Cyprinus carpio* diets on growth parameters, flesh quality, hematological profile, intestinal microbiota, oxidative status, and histological changes. Regarding growth parameters, BSFLM inclusion at 20% and 30% levels led to statistically significant improvements in FBW and WG compared to controls, with our results superior to those reported by [41] (62.20–64.58 and 26.90–26.35) or [28] (106.86–111.33 and 204.91–220.10). Condition factor values were close without significant differences, indicating good health status of studied specimens, with values superior to those obtained by [42] in *Micropterus salmoides* (1.15–1.42) or [43] in *Oreochromis niloticus* (1.57–1.62). FCR showed no statistical differences; however, treatments T3 and especially T4 demonstrated slight improvements, with values comparable to those recorded by [44] in *Salmo salar* (0.77–0.80), [45] in *Oncorhynchus mykiss* (0.89–2.72), [46] in *Sparus aurata* (1.12), and [42] in *Micropterus salmoides* (2.72–4.54). SGR values were high (3.45 ± 0.17–4.36 ± 0.11) compared to those in other studies [9,11,22,43] (0.75–0.79; 0.83–0.86; 2.17–2.40; 1.29–1.41). LER values showed a differences between T4 and the control (T1), indicating reduced lipid utilization, though values were comparable to other studies [9] (3.34–3.49). PER values were high in BSFLM treatments, demonstrating efficient protein utilization, with values slightly higher than those reported by [9,22] (1.68–1.76 and 2.08–2.10). VSI and HSI showed no statistical differences, with lower values compared to other studies [26,28,29] (7.72–8.05, 12.53–13.47, 9.04–9.41; 1.05–1.12, 1.27–1.29, 1.93). The positive results for the growth of carp at 20% and 30% BSFLM inclusion levels in this study may be attributed to the nutrient composition of BSFLM. According to [47], insect meal diets, including Black Soldier Fly, are rich in essential amino acids such as isoleucine, leucine, and lysine, which are critical for growth and development. Moreover, BSFLM is known to improve mineral content, providing vital elements like zinc, iron, and copper, which further support fish health and growth. These findings are consistent with [48], who highlighted the role of BSFLM in reducing aflatoxins, thus making it a safer and more sustainable alternative to traditional fish meal in aquaculture. Additionally, studies by [49] have shown that BSFLM contain valuable omega-3 fatty acids like Eicosapentaenoic acid (EPA) and Docosahexaenoic acid (DHA), which contribute to improved fish body composition. Reference [50] also found that substituting 30% of fish meal with insect larvae meals did not significantly affect growth performance in European sea bass, but there was a slight improvement in the feed conversion ratio when *Tenebrio molitor* was included. Furthermore, they observed variations in fatty acid profiles, with fish fed *Tenebrio molitor* diets having higher levels of n-6 polyunsaturated fatty acids compared to those fed *Hermetia illucens* and fish meal diets, which were rich in n-3 polyunsaturated fatty acids.

In humans, protein metabolism is strongly dependent on the intestinal microbiome, which is influenced by dietary components [51]. This principle is also applied to fish [52]. Protein-predominant dietary patterns stimulate proteolytic bacteria multiplication, which ferment undigested proteins and generate metabolites capable of modifying immune, metabolic, and neuronal responses [51,53]. In this study, supplementing the diet with 30% BSFLM in the T4 group favored sulfite-reducing Clostridia multiplication, which is unsurprising given these microorganisms’ affinity for protein-rich environments [54]. Long-term protein-rich diets leading to sulfite-reducing Clostridia proliferation can alter microorganism ratios and reduce total microorganism numbers [55], as observed in our study through decreased total aerobic bacteria. Increased sulfite-reducing Clostridia numbers correlated with elevated total anaerobic bacteria, which are essential for carbohydrate and amino acid fermentation necessary for organismal growth. Through compound metabolism, both Clostridia and other anaerobic microorganism groups, such as *Bacteroides species*, produce propionate, acetate, and especially butyrate, which limit pathogenic aerobic microorganism proliferation [56,57,58,59,60]. Although beneficial for undigested protein fermentation [51], Enterobacteriaceae family species multiplication predisposes individuals to digestive pathologies due to enterobacteria’s opportunistic pathogenic nature. While fermentative processes’ roles in fish are not fully elucidated, they are considered important for nutrient absorption, similar to other monogastric species [61].

The hematological examination revealed moderate changes, without significant variations. White blood cell population changes are normal consequences of intestinal microbiota variations related to feed composition. In this study, varying BSFLM concentrations introduced active molecules that exerted diverse effects on gut microorganisms, favoring certain bacterial species multiplication while inhibiting or bactericidally affecting others [62,63]. This mechanism causes aerobic and anaerobic microbial population ratio changes. Increased bacterial species numbers can induce intestinal white blood cell accumulation and influence inflammatory processes. Neutrophils are considered the most effective phagocytes responsible for extracellular pathogen elimination, while type 1 macrophages in intestinal walls serve as local sentinel cells providing backup systems against intracellular pathogens resistant to digestion [64]. Blood monocytes are tissue macrophage precursors, explaining observed value changes. Pathogen cell structure-dependent surface antigens (pathogen-associated molecular patterns) stimulate white blood cell recognition receptors. Enterobacteria, as Gram-negative cells with lipopolysaccharides in cell walls, stimulate cellular activation and pro-inflammatory cytokine release, attracting circulating cells and intensifying precursor cell differentiation, explaining monocytosis, lymphocytosis, or neutrophilia [65]. Similar studies on other fish species fed alternative food sources (sorghum, grape pomace, etc.) have highlighted feeding type importance and effects on intestinal bacterial populations [39,52,66]. Other factors influence microbial variability, including individual, seasonal, and growth pattern variations (cultivated/wild), developmental stage/age, etc., demonstrating that microbiota is dynamic and context-dependent, significantly limiting universal fish microbiota pattern establishment [61,67]. It should be noted that the microbiota assessment in this study was based on culture-dependent methods to capture the abundance of specific functional microbial groups (total aerobic and anaerobic bacteria, sulfite-reducing Clostridia, and Enterobacteriaceae). Therefore, future studies using culture-independent techniques such as 16S rRNA sequencing are warranted to provide a more comprehensive characterization.

Oxidative stress occurs when reactive oxygen species (ROS) production disproportionately exceeds antioxidant system capacity to control damaging effects. ROS are essential normal metabolic products that can damage nuclear DNA and harm cellular membrane proteins and lipids if not suppressed by antioxidant mechanisms [27,28]. According to [6,68], oxidative stress occurs when ROS production and tissue antioxidant scavenging capacity through antioxidant enzymes (SOD, CAT, GPX) become imbalanced, resulting in cellular membrane lipid damage. Lipid peroxidation values measure free radical lipid attack degrees [69]. Insect meal inclusion effects on aquafeed oxidative stress remain relatively unexplored, with some studies indicating enhanced antioxidant capacity [70] while others report opposite effects [68,71]. Antioxidant enzyme responses in BSFLM-containing diets vary considerably depending on species and analyzed tissues. The first line of ROS defense is the CAT-SOD enzyme mechanism, where SOD catalyzes superoxide anion reduction to hydrogen peroxide, subsequently decomposed by CAT at intra- and extracellular levels, with CAT activity correlated with H_2_O_2_ concentration increases [72]. In BSFLM-fed fish livers, SOD activity increased, underscoring this adaptive response’s significance and highlighting its important antioxidant defense role [6]. Increased hepatic and muscular SOD and CAT activities observed in BSFLM diets align with hepatic peroxidation intensification and suggest increased superoxide anion production. The highest MDA concentrations were recorded in liver tissue, consistent with literature data indicating significant dietary supplementation effects on hepatic MDA concentrations [73]. From a mechanistic perspective, the tissue-level responses observed in this study at higher BSFLM inclusion levels may be linked to alterations in redox homeostasis to the radical-scavenging activity of chitin and chitosan present in insect meals [27,74]. Chitin exhibits antioxidant capacity; therefore, potential BSF antioxidant presence may have mitigated oxidative stress, explaining lipid peroxidation reduction and SOD, CAT, and GPX activity changes [27]. Insects contain significant exoskeleton chitin amounts, with chitin and derivatives reported to possess antioxidant properties preventing deleterious disease effects [72,75]. Other *Hermetia illucens* diet studies observed no SOD, CAT, and GPX activity differences in rainbow trout liver and kidney [34], while plasma CAT activity increased in Jian carp [72]. Increased SOD and CAT activities were observed with cricket meal dietary inclusion in African catfish [27,76]. SOD activity increased in rainbow trout liver [45] and snakehead fed *Hermetia illucens*-containing diets [25,27]. Other data showed CAT activity increases in carp liver when including maggot meal [77]. The intestine, as the first food contact barrier, is typically more susceptible to oxidative stress [74,78]. Different liver and intestinal antioxidant responses were reported for meager and pikeperch fed insect meal-containing diets [27,79]. Unlike liver and muscle observations, intestinal antioxidant enzyme activity was not significantly affected by experimental diets.

The histological results obtained following examination of the intestine and hepatopancreas *in Cyprinus carpio* specimens subjected to experimental diets with BSFLM demonstrate a variable degree of tissue adaptation depending on the level of fishmeal replacement. In the control group, the histological structure of both the intestine and hepatopancreas was preserved, reflecting a normal physiological state. The intestinal villi presented uniform height and well-organized simple columnar epithelium, while at the hepato-pancreatic level, regular hepatocyte cords and well-differentiated pancreatic acini were observed. This aspect is consistent with the specialized literature, which describes this diet as optimal for teleost fish growth [80]. In T2 (10% BSFLM), no significant structural modifications were observed. The intestine presented well-developed villi with normal epithelial organization, while hepatic tissue maintained a compact architecture with viable hepatocytes and intact pancreatic acini. The slight cytoplasmic vacuolization observed at the hepatic level can be interpreted as transitory metabolic adaptation modifications without pathological implications. These findings suggest that moderate replacement of fishmeal with insect meal is well-tolerated by carp, without affecting the histological integrity of digestive organs [35]. In T3 (20% BSFLM), the observed histological modifications were minimal, while the tissue architecture of both the intestine and hepatopancreas remained well-preserved. The intestinal villi presented a robust appearance, and the crypts of Lieberkühn showed moderate deepening, compatible with intensified proliferative activity. In the hepatopancreas, pancreatic acinar structure was well-represented, and hepatocytes presented intense metabolic activity without signs of degeneration. Our results are consistent with recent studies indicating that the use of insect larvae (e.g., *Hermetia illucens*) at moderate inclusion levels does not adversely affect the anatomical integrity of the intestine and hepatopancreas, while maintaining normal tissue organization and cellular morphology [12,36]. In T4 (30% BSFLM), specimens presented more pronounced histological modifications, which may indicate functional stress at both intestinal and hepatic levels. At the intestinal level, shorter and sometimes thickened villi were noted, with a more compact architecture and crypts with hyperplastic appearance. These modifications may reflect a compensatory reaction of the intestinal mucosa, possibly generated by changes in dietary composition or nutrient digestibility. At the hepato-pancreatic level, cytoplasmic vacuolization and sparser distribution of pancreatic acini may signal increased metabolic load, compatible with limited tolerance to this proportion of insect meal. The present findings, showing improved growth performance at the highest BSFLM inclusion level alongside histological alterations, are consistent with previous studies reporting enhanced growth at moderate-to-high inclusion levels of insect meal in carp diets. For example, Dogan et al. (2021) [31] reported improved growth performance at intermediate inclusion levels, while higher inclusion rates may induce intestinal stress and histopathological alterations. Similarly, Albayati et al. (2025) [32] observed significant improvements in growth performance and feed efficiency at high inclusion levels of BSFLM in common carp. In contrast, Zhou et al. (2017) [30] found that complete replacement of fishmeal did not significantly affect growth performance but induced changes in lipid metabolism and fatty acid composition, suggesting underlying physiological adjustments. Additionally, the chitin content of insect meal may reduce digestibility and induce mild intestinal irritation, contributing to villi structural changes. Furthermore, the increased antioxidant enzyme activity observed at high inclusion levels suggests the activation of compensatory defense mechanisms in response to elevated oxidative stress. This adaptive response may temporarily maintain physiological homeostasis despite underlying tissue alterations. Together with potential microbiota shifts, these findings indicate that while high BSFLM inclusion enhances growth performance, it may exceed the optimal physiological tolerance threshold, resulting in subclinical tissue stress. Previous studies have reported similar effects when inclusion levels exceeded 25–30%, with impact on hepatic function and villous structure [81,82]. These changes, at the 30% inclusion level, suggest functional stress, indicating limited tolerance to the higher inclusion level. The anatomical effects observed at the intestinal and hepatic levels appear to be species-specific. For example, in pikeperch (*Sander lucioperca*) fed *Hermetia illucens* (HI) diets, no significant morphological changes were observed in the intestine, although a trend for wider villi was noted in the HI36 group (*p* = 0.065) [79]. This suggests that HI inclusion does not negatively affect the intestinal development of pikeperch. Mild-to-severe liver vacuolar degeneration was recorded in all groups, with greater severity in the control and HI9 (9%) groups. However, no inflammation was observed in the liver, indicating that dietary HI did not cause liver inflammation. Species-dependent responses have also been reported in other fish. No anatomical alterations were observed in *Acipenser baerii*, although changes were noted in oxidative stress biomarkers, whereas in Nile tilapia, dietary HI was associated with improved hepatic parenchymal organization [83,84]. The histological data support the idea that moderate levels of fishmeal substitution with insect meal can be utilized in carp feed without negative morphological effects, and even with possible functional benefits. At 30% inclusion, histological signs of adaptive stress appear, which could limit long-term benefits.

## 5. Conclusions

Dietary inclusion of Black Soldier Fly Larvae Meal (BSFLM) in two-year-old common carp enhanced growth performance, with 30% inclusion increasing final body weight by 17% and weight gain by 23% compared to the control. Flesh protein remained stable, while fat, ash, and collagen decreased at higher inclusion levels. Hematological parameters were largely unaffected, indicating no adverse effects on blood health. Intestinal microbiota responded selectively, with anaerobic bacteria increasing proportionally with BSFLM, and antioxidant enzyme activities improved, reducing lipid peroxidation. Overall, BSFLM supports growth and gut–liver function, with 10–20% inclusion appearing optimal to balance performance and tissue health.

## Figures and Tables

**Table 1:** Water parameters during the feed trial.

Parameter	Range	Reference Values ^1^
Temperature (°C)	20.50–25.20	22–26
pH	7.80–8.00	6.50–8.50
Dissolved oxygen (mg L^−1^)	7.99–8.65	>5.00
Total ammonia (mg L^−1^)	0.00–0.05	<0.01
Ammonium (mg L^−1^)	0.00–0.06	≤0.50
Nitrate (mg L^−1^)	3.30–10.30	0–400
Nitrite (mg L^−1^)	0.06–0.10	<0.10
Total hardness (mg L^−1^)	112–138	50–400
Calcium (mg L^−1^)	89–106	4–160
Magnesium (mg L^−1^)	70–90	<15
Iron (mg L^−1^)	0.00–0.03	<0.15

^1^ Reference values according to Timmons, 2022 [37].

**Table 2:** Chemical composition of ingredients (%).

Ingredients	Moisture (%)	Crude Protein (%)	Crude Fat (%)	Crude Fiber (%)
Meat meal	7.13	40.00	5.00	-
Fish meal	9.57	53.00	3.50	-
Soybean meal	12.5	42.5	1.8	4.63
BSFLM	3.80	42.02	9.49	-
Peas	13.66	26.83	7.83	3.54
DDGS	10.47	22.51	9.70	8.17
Sunflower meal	11.10	32.00	0.71	19.08
Soybean oil	0.00	0.00	91.90	0.00
Wheat	13.91	11.98	1.19	2.70
Corn	12.58	9.19	2.22	1.93

**Table 6:** Growth performance parameters of common carp fed BSFLM experimental diets.

Parameter		Treatment			Anova	Polynomial Contrasts	
	T1 (0% BSFLM)	T2 (10% BSFLM)	T3 (20% BSFLM)	T4 (30% BSFLM)	*p*-Value ^1^	Linear	Quadratic
IBW (g)	107.84 ± 2.79	115.1 ± 2.53	109.76 ± 1.78	115.56 ± 2.04	0.05	0.08	0.75
FBW (g)	297.63 ± 10.42 ^bc^	265.14 ± 8.26 ^c^	321.28 ± 9.58 ^ab^	349.15 ± 10.79 ^a^	0.001	0.001	0.003
WG (g)	189.79 ± 2.14 ^bc^	152.98 ± 7.29 ^c^	208.57 ± 4.23 ^ab^	233.6 ± 13.86 ^a^	0.001	0.001	0.005
FCR	2.28 ± 0.08	2.22 ± 0.06	2.16 ± 0.05	2.12 ± 0.04	0.307	0.073	0.991
SGR (% day^−1^)	4.01 ± 0.08 ^ab^	3.45 ± 0.17 ^b^	4.09 ± 0.13 ^a^	4.36 ± 0.11 ^a^	0.006	0.5	0.003
RGR (g g^−1^ day^−1^)	0.18 ± 0.04	0.14 ± 0.03	0.18 ± 0.05	0.21 ± 0.06	0.72	0.79	0.55
PER	0.76 ± 0.02	0.77 ± 0.02	0.86 ± 0.02	0.82 ± 0.01	0.037	0.03	0.084
LER	3.11 ± 0.1 ^ab^	3.46 ± 0.09 ^a^	3.13 ± 0.09 ^ab^	2.99 ± 0.05 ^b^	0.021	0.936	0.01
CF	1.88 ± 0.03	1.82 ± 0.04	1.76 ± 0.03	1.75 ± 0.04	0.05	0.005	0.551
HSI (%)	0.27 ± 0.03	0.24 ± 0.03	0.21 ± 0.03	0.28 ± 0.02	0.372	0.599	0.109
VSI (%)	9.98 ± 0.75	10.76 ± 1.13	11.59 ± 1.13	11.1 ± 1.09	0.74	0.593	0.552

Different lowercase letters represent statistically significant differences according to Tukey’s test at *p* < 0.05. Values are presented as mean ± standard error. ^1^ One-way ANOVA. Abbreviations: IBW (initial body weight); FBW (final body weight); WG (weight gain); CF (condition factor); FCR (feed conversion ratio); SGR (specific growth rate); RGR (relative growth rate); PER (protein efficiency ratio); LER (lipid efficiency ratio); HIS (hepatosomatic index), VSI (viscerosomatic index).

**Table 7:** Biochemical composition of common carp flesh fed with BSFLM experimental diets.

Treatment	Parameter					
	Moisture (%)	Protein (%)	Fat (%)	Ash (%)	Collagen (%)	Salt (%)
T1 (0% BSFLM)	74.66 ± 0.22 ^b^	18.4 ± 0.19	2.16 ± 0.11 ^a^	1.7 ± 0.11 ^b^	0.8 ± 0.09 ^b^	0.54 ± 0.19
T2 (10% BSFLM)	75.6 ± 0.44 ^ab^	18.4 ± 0.09	2.3 ± 0.11 ^a^	2.34 ± 0.19 ^b^	1.18 ± 0.09 ^a^	1.06 ± 0.08
T3 (20% BSFLM)	76.48 ± 0.65 ^a^	18.8 ± 0.21	0.92 ± 0.17 ^c^	3.94 ± 0.32 ^a^	0.88 ± 0.09 ^ab^	1.04 ± 0.2
T4 (30% BSFLM)	76.6 ± 0.32 ^a^	18.84 ± 0.18	1.5 ± 0.13 ^b^	0.88 ± 0.06 ^c^	0.34 ± 0.07 ^c^	0.46 ± 0.18
*p*-value	0.022	0.158	0.001	0.001	0.001	0.039

Different lowercase letters represent statistically significant differences according to Tukey’s test at *p* < 0.05. Values are presented as mean ± standard error.

**Table 8:** Hematological parameters of carp under different dietary treatments.

Parameter	T1 (0% BSFLM)	T2 (10% BSFLM)	T3 (20% BSFLM)	T4 (30% BSFLM)	*p*-Value ^1^
WBC (×10^9^/L)	47.70 ± 4.13	52.09 ± 0.38	51.12 ± 0.34	50.40 ± 1.55	0.59
LYM (×10^9^/L)	44.01 ± 4.24	41.88 ± 5.11	43.65 ± 0.93	46.37 ± 2.28	0.492
MON (×10^9^/L)	0.45 ± 0.19	2.59 ± 2.31	0.26 ± 0.01	0.26 ± 0.01	0.13
NEU (×10^9^/L)	2.55 ± 0.22	7.43 ± 2.23	5.31 ± 0.52	3.20 ± 0.61	0.13
EOS (×10^6^/L)	0.30 ± 0.05	0.38 ± 0.02	0.26 ± 0.01	0.24 ± 0.08	0.321
BAS (×10^9^/L)	0.03 ±0.00	0.02 ± 0.00	0.02 ± 0.01	0.02 ± 0.01	0.803
RBC (×10^12^/L)	1.35 ± 0.18	1.48 ± 0.23	1.38 ± 0.05	1.50 ± 0.29	0.941
HGB (g/dL)	15.85 ± 0.49	15.40 ± 0.83	17.50 ± 0.49	15.28 ± 0.69	0.105
HCT (%)	13.28 ± 1.76	14.53 ± 2.32	13.61 ± 0.48	14.91 ± 3.04	0.94
MCV (fl)	98.50 ± 1.32	98.25 ± 0.63	99.00 ± 2.04	98.75 ± 2.43	0.991
PLT (×10^9^/L)	34.75 ± 2.17	31.50 ± 4.01	38.75 ± 7.04	37.5 ± 5.98	0.752
MPV (fl)	10.25 ± 0.50	10.28 ± 0.24	10.40 ± 0.29	9.48 ± 0.09	0.206
PCT (%)	0.04 ±0.00	0.03 ± 0.00	0.04 ± 0.01	0.04 ± 0.01	0.941

Values are presented as mean ± standard error. ^1^ One-way ANOVA. Abbreviations: WBC (white blood cell); LYM (lymphocytes); MON (monocytes); NEU (neutrophils); EOS (eosinophils); BAS (basophils); RBC (red blood cell); HGB (hemoglobin); HCT (hematocrit); MCV (mean corpuscular volume); PLT (platelets); MPV (mean platelet volume); PCT (plateletcrit).

## Data Availability

The original contributions presented in this study are included in the article. Further inquiries can be directed to the corresponding authors.

## Abbreviations

BSFLM	Black Soldier Fly Meal
WBC	Leukocytes
RBCs	Red Blood Cells
HGB	Hemoglobin
HCT	Hematocrit
PCT	Thrombocrit
SOD	Superoxide dismutase
CAT	Catalase
GPX	Glutathione peroxidase
